# Viewing Instructions Accompanying Action Observation Modulate Corticospinal Excitability

**DOI:** 10.3389/fnhum.2016.00017

**Published:** 2016-02-01

**Authors:** David J. Wright, Sheree A. McCormick, Jacqueline Williams, Paul S. Holmes

**Affiliations:** ^1^Centre for Health, Exercise and Active Living, Manchester Metropolitan UniversityUK; ^2^College of Sport and Exercise Science, Institute of Sport, Exercise and Active Living, Victoria UniversityMelbourne, VIC, Australia

**Keywords:** combined observation and imagery, action observation, movement imagery, transcranial magnetic stimulation, motor (re)learning

## Abstract

Action observation interventions may have the potential to contribute to improved motor function in motor (re)learning settings by promoting functional activity and plasticity in the motor regions of the brain. Optimal methods for delivering such interventions, however, have yet to be established. This experiment investigated the effect on corticospinal excitability of manipulating the viewing instructions provided to participants (*N* = 21) prior to action observation. Specifically, motor evoked potential responses measured from the right hand muscles following single-pulse transcranial magnetic stimulation (TMS) to the left motor cortex were compared when participants were instructed to observe finger-thumb opposition movement sequences: (i) passively; (ii) with the intent to imitate the observed movement; or (iii) whilst simultaneously and actively imagining that they were performing the movement as they observed it. All three action observation viewing instructions facilitated corticospinal excitability to a greater extent than did observation of a static hand. In addition, the extent to which corticospinal excitability was facilitated was greater during combined observation and imagery, compared to passive observation. These findings have important implications for the design of action observation interventions in motor (re)learning settings, where instructions that encourage observers to simultaneously imagine themselves performing the observed movement may offer the current optimal method for improving motor function through action observation.

## Introduction

Several neural areas active during physical execution of movement, including premotor cortex, primary motor cortex, supplementary motor area and superior parietal lobe, are also active during observation of similar movements (e.g., Buccino et al., 2001; Grèzes and Decety, 2001; Filimon et al., 2007). Accordingly, action observation interventions may contribute to improved motor function by promoting topographic cortical activity and plasticity in the motor regions of the brain. This mechanism for motor (re)learning through observation has been advocated in both sport (Holmes and Calmels, 2008) and clinical (Ertelt and Binkofski, 2012) domains. The optimal methods for delivering action observation interventions are, however, currently unknown. For example, action observation interventions could be viewed: (i) passively, without any additional instructions; (ii) with the intent to subsequently imitate the observed movements; or (iii) whilst simultaneously and actively imagining performing the movements. Identifying which approach to action observation produces the strongest activity in motor regions of the brain will add to the understanding of action observation interventions and their delivery in motor (re)learning settings.

Transcranial magnetic stimulation (TMS) is one method that has been used extensively to study the effect of action observation on activity in the human motor system. When single-pulse TMS is applied to the primary motor cortex, motor evoked potentials (MEPs) are elicited in the corresponding contralateral muscles. The amplitude of the resulting MEPs, measured by electromyography (EMG), provides a measure of corticospinal excitability at the time of stimulation (Rothwell, 1997; Naish et al., 2014). The first study to use TMS to investigate corticospinal excitability during action observation was conducted by Fadiga et al. (1995). These researchers demonstrated that when single-pulse TMS was applied to the hand representation of the left motor cortex during passive observation of hand movements (e.g., reaching and grasping objects, air-tracing letters of the Greek alphabet), the amplitude of the resulting MEPs were greater than those obtained during observation of static objects. In addition, this facilitation in MEP amplitude during action observation was specific to the muscles that would have been involved in performing the observed actions. Since Fadiga et al.’s experiment, the finding of increased corticospinal excitability during action observation has been replicated extensively (e.g., Strafella and Paus, 2000; Gangitano et al., 2001; Patuzzo et al., 2003; Borroni et al., 2005; Loporto et al., 2012; Williams et al., 2012; for reviews, see Loporto et al., 2011 and Naish et al., 2014). Collectively, these results indicate that passive observation (i.e., observation with no additional viewing instructions) of human movement can facilitate corticospinal excitability, supporting the use of action observation as a motor (re)learning technique.

The effect of a facilitation in corticospinal excitability during action observation is usually attributed to activity of the putative human mirror neuron system (hMNS). First identified in the Macaque monkey by di Pellegrino et al. (1992), mirror neurons are active during both execution and observation of movements. Neurons with similar properties have been proposed to exist in the premotor cortex and inferior parietal lobule of the human brain (Rizzolatti and Craighero, 2004), and the first direct evidence of this was provided by Mukamel et al. (2010). The facilitation of corticospinal excitability following stimulation of the primary motor cortex during action observation experiments using TMS is generally accepted to be caused by increased activity in premotor areas of the hMNS, which connect to the primary motor cortex via strong cortico-cortical connections (Fadiga et al., 2005).

Although the corticospinal excitability facilitation effect during passive observation is well-established, some researchers have examined whether providing viewing instructions that encourage better engagement in the observation process can produce a stronger facilitation of corticospinal excitability. One strategy that has received research attention is to instruct participants to observe the movement with the intent to imitate it, rather than observe passively. Research exploring this issue using TMS techniques has produced equivocal results (e.g., Clark et al., 2004; Roosink and Zijdewind, 2010; Hardwick et al., 2012). In the first TMS study to compare these two viewing conditions, Clark et al. (2004) examined MEP amplitudes obtained when participants observed passively a scissor movement made by the index and middle fingers, or observed with the intent to imitate an “OK” sign made by the thumb and index finger. Although both types of observation produced MEPs of larger amplitude than control conditions, there was no difference in MEP amplitude between the passive observation and observe to imitate conditions. This finding should be interpreted cautiously as two different types of movement task were used, making it difficult to compare accurately the results of the two conditions. In a similar experiment by Hardwick et al. (2012), results indicated that whilst passive observation of grasping and finger abduction-adduction movements facilitated corticospinal excitability in comparison to a control condition involving observation of a fixation cross, no such facilitation occurred when participants observed with the intent to imitate the same movements. To explain this finding, the authors argued that the increased activity found consistently during observe to imitate conditions in fMRI studies (e.g., Iacoboni et al., 1999; Buccino et al., 2004) may be representative of inhibitory mechanisms acting to prevent overt imitation of the observed task, represented as suppression of MEP amplitude. Although this explanation seems plausible, the findings of Roosink and Zijdewind’s (2010) TMS experiment conflict with this explanation. They measured MEP amplitudes during observation of finger tapping sequences; more novel movements than those used by Clark et al. (2004) and Hardwick et al. (2012). Facilitation of corticospinal excitability was larger in an observe to imitate condition, compared to a passive observation condition. This led Roosink and Zijdewind to suggest that instructing participants to observe with the intent to imitate the movement may be the most appropriate instruction to provide when using observation interventions in motor (re)learning settings. Given these conflicting findings, however, further research is required to establish the effect on corticospinal excitability of instructing participants to observe with the intent to imitate during action observation.

Another strategy that has been used in an attempt to increase facilitation of corticospinal activity during action observation is to instruct participants to imagine that they are performing the movement whilst they observe it. As both observation and imagery appear to activate the motor system in a similar manner (e.g., Grèzes and Decety, 2001; Jeannerod, 2001; Clark et al., 2004; Filimon et al., 2007; Munzert et al., 2008; Williams et al., 2012), engaging simultaneously in the two techniques may produce stronger activity in the motor regions of the brain than either technique in isolation (for a review, see Vogt et al., 2013). Sakamoto et al. (2009) were the first to investigate this idea using TMS. They reported that combined observation and imagery of a bicep curl movement produced MEPs of larger amplitude than those obtained when the same movement was observed or imaged in isolation. This finding has since been replicated in several other experiments (e.g., Ohno et al., 2011; Tsukazaki et al., 2012; Wright et al., 2014; Mouthon et al., 2015). Collectively, this research indicates that combined observation and imagery of a variety of upper limb movement tasks produces greater facilitation of corticospinal excitability than occurs during passive observation. As such, these authors have argued that combined observation and imagery may offer the current optimal method for delivering observation interventions in motor (re)learning settings, although these claims may be premature given that no experiment has yet compared the effect of observe to imitate instructions against combined observation and imagery instructions.

Despite considerable TMS research into the effects of passive observation, observation with the intent to imitate, and combined observation and imagery, no study has yet compared directly the effects of these three observation instructional strategies on corticospinal excitability in a single experiment. The aim of this study was, therefore, to determine whether providing different viewing instructions prior to action observation would modulate corticospinal excitability in healthy participants. Specifically, the experiment aimed to compare MEP amplitudes obtained during passive observation, observation with the intent to imitate, or combined observation and imagery of novel finger-thumb opposition movement sequences. It was hypothesized that: (i) all three movement observation conditions would facilitate corticospinal excitability in comparison to a control condition involving observation of a static hand and (ii) the facilitation of corticospinal excitability obtained during both the combined observation and imagery and observation with the intent to imitate conditions would be greater than that obtained during passive observation. The predicted facilitation of corticospinal excitability during action observation was, however, only expected in the muscles that would be involved in the observed action at the time of stimulation. No prediction was made for differences in corticospinal excitability between the combined observation and imagery and observation with the intent to imitate conditions.

## Materials and Methods

### Participants

Twenty-one healthy volunteers (13 male, 8 female) aged 20–31 years (mean age 23.1 ± 3.16 years) participated in the experiment. Seventeen participants were right-handed and four were left-handed, as assessed by the Edinburgh Handedness Inventory (Oldfield, 1971), and all participants had normal or corrected-to-normal vision. All participants were naïve to the purpose of the experiment. Prior to participation, the TMS Adult Safety Screen (Keel et al., 2001) was used to identify any participants who may have been predisposed to adverse effects of the stimulation. No participants were excluded from the experiment based on their responses to this questionnaire and no adverse responses to the stimulation were reported during the experiment. All participants provided their written informed consent to take part in the experiment and ethical approval was granted by the University Ethics Committee at the host institution. The experiment was conducted in accordance with the Declaration of Helsinki.

### Electromyography Recording and Transcranial Magnetic Stimulation Procedure

Electromyography (EMG) was recorded simultaneously from the opponens pollicis (OP), first dorsal interosseous (FDI) and abductor digiti minimi (ADM) muscles of the right hand using a Delsys Bagnoli 8 EMG System (Delsys Inc., Boston, MA, USA) and DE-2.1 bipolar, single differential, surface EMG electrodes (Delsys Inc). All electrode sites were cleaned with an alcohol wipe prior to electrode attachment and electrodes were placed over the mid-point of the belly of the muscles. A reference electrode was positioned over the ulnar process of the right wrist. The EMG signal was recorded using Spike 2 version 6.18 software (Cambridge Electronic Design, Cambridge, UK) with a sampling rate of 2 kHz, bandwidth of 20 Hz to 450 kHz, 92 dB common mode rejection ratio and >10^15^ Ω input impedance, received by a Micro 1401-3 analog-to-digital converter (Cambridge Electronic Design).

Single-pulse TMS was delivered using a figure-of-eight coil (two 70 mm diameter loops) connected to a Magstim 200^2^ magnetic stimulator (Magstim Co., Whitland, Dyfed, UK), which delivered monophasic pulses with a maximum field strength of 2.2 Tesla. The coil was held in a fixed position against the scalp by a mechanical arm over the hand representation of the left motor cortex. The coil was orientated at a 45° angle to the central line between nasion and inion landmarks to induce current flow in the brain in a posterior-anterior direction and perpendicular to the central sulcus. This orientation was used as it is the optimal orientation for achieving indirect trans-synaptic activation (Brasil-Neto et al., 1992). The optimal scalp position (OSP) was identified as the scalp location that produced MEPs of the largest amplitude in the OP muscle, whilst also eliciting consistent MEPs from the FDI and ADM muscles, using a stimulation intensity of 60% maximum stimulator output. The use of 60% maximum stimulator output as the intensity for finding the OSP was selected as it produces consistently large amplitude MEPs in most individuals and is consistent with the procedure used for finding the OSP in other TMS action observation experiments (e.g., Clark et al., 2004; Williams et al., 2012; Wright et al., 2014). To ensure a consistent coil positioning throughout the experiment, the OSP was marked on a tightly fitting polyester cap worn by the participants and the coil position was continuously checked against this mark throughout the experiment. Each participant’s resting motor threshold (RMT) was determined by gradually reducing or increasing the stimulation intensity to find the minimum intensity capable of eliciting peak-to-peak MEP amplitudes from the OSP in excess of 50 μV in 5 out of 10 trials (Rossini et al., 1994, 2015). RMT values ranged from 38–57% maximum stimulator output and the stimulation intensity for the experiment was set at 110% RMT. This intensity was chosen based on Loporto et al.’s (2013) finding that facilitation of corticospinal excitability during action observation is more likely to occur via indirect wave stimulation at 110% RMT compared to higher stimulation intensities.

### Procedure

Participants were seated at a table in a dimly lit room. Their elbows were flexed at approximately 90° and their hands rested on the table with the palms facing upwards. The participant’s head was positioned in an adjustable head-and-chin rest to restrict movement. A 32-inch LCD TV screen (DGM Model LTV-3203H) was positioned in a custom-built stand at an angle of 15° to the table and positioned over the participants’ hands to obscure them from view. Blackout curtains were drawn along either side of the table and behind the screen to remove any distracting visual stimuli from the participant’s field of vision. Participants were asked to avoid voluntary movement during the experiment and to follow the viewing instructions provided prior to each experimental condition.

**Figure 1:**
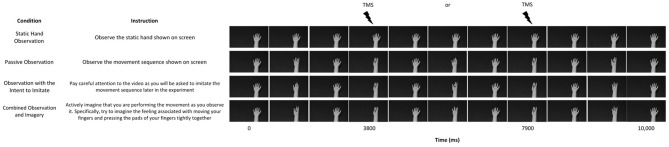
**A schematic representation of the four conditions in the experiment.** All videos were 10,000 ms duration and one stimulation was delivered per trial at either 3800 or 7900 ms.

For methodological reasons discussed below, participants took part in four fixed order conditions: static hand observation, passive observation, observation with the intent to imitate, and combined observation and imagery (see Figure 1). The static hand condition showed the palmar view of a still right hand. For this condition, participants were instructed to “observe the static hand shown on screen”. The passive observation condition showed the palmar view of a right hand performing a sequence of finger-thumb opposition movements (middle-little-index-little-ring) at a frequency of 0.6 Hz. For this condition, participants were instructed to “observe the movement sequence shown on screen”. The observe with the intent to imitate condition showed the same hand in the same position, performing a different sequence of finger-thumb opposition movements (index-little-ring-little-middle) at a frequency of 0.6 Hz. For this condition, participants were instructed to: “observe the movement closely as you will be asked to imitate the movement sequence later in the experiment”. Finally, the combined observation and imagery condition showed the same hand in the same position, performing another different sequence of finger-thumb opposition movements (ring-little-middle-little-index) at a frequency of 0.6 Hz. For this condition, participants were instructed to “actively imagine that you are performing the movement as you observe it. Specifically, try to imagine the feeling associated with moving your fingers and pressing the pads of your fingers tightly together”. Participants were instructed to focus specifically on kinaesthetic imagery (i.e., imagining the physiological sensations associated with performing the movement) as Stinear et al. (2006) have demonstrated that this type of imagery facilitates corticospinal excitability to a greater extent than visual imagery. In an attempt to make sure that participants did not focus on previous viewing instructions during subsequent experimental conditions, participants were instructed prior to each block that they would be starting a new experimental condition and should focus only on the viewing instructions provided for that specific block.

Conditions were presented in a fixed order, as opposed to being randomized across the experiment, for two reasons. First, and consistent with Roosink and Zijdewind (2010), presenting the observe to imitate condition after the passive observation condition was important to reduce the chances of participants engaging in their observe to imitate strategy during passive observation trials. Second, and consistent with Wright et al. (2014), presenting the combined observation and imagery condition at the end of the experiment was important to reduce the likelihood of participants actively engaging in movement imagery during static hand, passive observation, or observe to imitate trials. In addition, a different sequence of finger-thumb opposition movements was used for the three experimental conditions to ensure that the stimulus viewing time and stimulus novelty remained constant across conditions.

The videos, filmed from an egocentric perspective, showed the hand positioned to the right of the screen so that the hand in the video appeared directly over the participant’s own right hand, using a flat screen projection. The purpose of this was to create a more embodied perspective of the hand than would have been possible with the typical vertical screen angle. The hand in the videos showed a neutral hand of the same sex and skin tone as the participant’s (i.e., male participants observed a male hand of similar skin tone to their own, and female participants observed a female hand of similar skin tone to their own). There were 24 trials for each condition, and one stimulation was delivered per trial. All videos were of 10 s duration, and the timing of the stimulation was varied to occur either 3800 or 7900 ms after the onset of the video to avoid the participant’s anticipation of the stimulations. Both stimulation timings corresponded to the point of contact between the thumb and little finger (maximum thumb opposition) in the three experimental conditions, stimulations being matched across conditions irrespective of finger touch sequence. Participants were given a 2 minute rest period between each experimental condition. Prior to each condition, six stimulations were delivered to all participants whilst observing a black fixation cross on a white screen. The purpose of this was to control for any coil movement between the conditions.

### Data Analysis

The peak-to-peak amplitude of EMG activity 200 ms prior to each stimulation was measured. As heightened EMG activity pre-stimulation may influence the amplitude of the subsequent MEP, any MEPs in which the pre-stimulation peak-to-peak EMG amplitude was 2.5 *SD* higher than the mean baseline EMG amplitude of each participant for that muscle were discarded from further analysis. To confirm that there were no differences in the number of rejected MEPs across muscles and experimental conditions, the number of rejected MEPs per muscle and condition was analyzed using a 3 (muscle) × 4 (condition) repeated measures ANOVA. In addition, to confirm that there were no differences in the pre-stimulation EMG activity across muscles and experimental conditions, the amplitude values for the pre-stimulation EMG in the remaining trials were then submitted to a 3 (muscle) × 4 (condition) repeated measures ANOVA.

Due to the blocked nature of the experiment, it was possible that minor changes in coil positioning following rest periods between conditions could have influenced the amplitude of the MEPs obtained in some conditions. To confirm that any possible changes in coil position across blocks did not influence the results, a 3 (muscle) × 4 (condition) repeated measures ANOVA was performed on the mean MEP amplitudes of the six fixation cross trials observed immediately prior to each experimental condition.

For the main analysis, peak-to-peak MEP amplitude values were extracted from the EMG data for each individual trial and averaged for each condition. Given the inherent variability in MEP amplitudes between trials and between participants (Kiers et al., 1993), MEP data was normalized using the *z*-score transformation common in TMS action observation research (e.g., Fadiga et al., 1995; Roosink and Zijdewind, 2010; Loporto et al., 2013; Wright et al., 2014). Following *z*-score computation models of Loporto et al. (2013), *z*-scores were calculated by muscles relative to all conditions (e.g., *z*-score values for MEPs in the FDI muscle were calculated based on MEP values obtained in this muscle in all experimental conditions). Normalized MEP amplitudes were then analyzed using a 3 (muscle) × 4 (condition) repeated measures ANOVA. *Post hoc* analyses with the Bonferroni adjustment were applied where significant effects were reported. Where Mauchley’s test indicated that the assumption of sphericity had been violated, the degrees of freedom were corrected using the Greenhouse-Geisser method. The alpha level for statistical significance for all analyses was set at *p* = 0.05, and effect sizes are reported as partial eta squared (${\text{η}}_{\rho }^{2}$). All statistical analyses were conducted using the IBM SPSS Statistics 21 software.

## Results

### Number of Rejected Trials

The mean number of rejected MEPs per muscle per condition were less than two across all testing conditions. The 3 (muscle) × 4 (condition) repeated measures ANOVA performed on the number of rejected MEPs data showed no significant main effects for muscle, *F*_(2,40)_ = 0.79, *p* = 0.46, ${\text{η}}_{\rho }^{2}$ = 0.04 or condition, *F*_(3,60)_ = 0.50, *p* = 0.68, ${\text{η}}_{\rho }^{2}$ = 0.03. In addition, there was no significant muscle × condition interaction, *F*_(6,120)_ = 0.49, *p* = 0.82, ${\text{η}}_{\rho }^{2}$ = 0.02. This confirmed that the number of MEPs rejected due to high baseline EMG activity did not differ between muscles or conditions.

### Baseline EMG Data

The 3 (muscle) × 4 (condition) repeated measures ANOVA performed on the pre-stimulation EMG amplitudes showed no significant main effects for muscle, *F*_(1.5,30.5)_ = 0.87, *p* = 0.40, ${\text{η}}_{\rho }^{2}$ = 0.04 or condition, *F*_(2.3,45.1)_ = 1.84, *p* = 0.17, ${\text{η}}_{\rho }^{2}$ = 0.08. In addition, there was no significant muscle × condition interaction, *F*_(3.4,67.4)_ = 2.01, *p* = 0.11, ${\text{η}}_{\rho }^{2}$ = 0.09. This confirmed that any facilitation in MEP amplitude could not be attributed to increased EMG activity at the time of stimulation in certain conditions.

### Pre-Condition Fixation Cross Data

The 3 (muscle) × 4 (condition) repeated measures ANOVA performed on the pre-condition fixation cross MEP amplitudes showed no significant main effects for muscle, *F*_(1.4,27.3)_ = 1.32, *p* = 0.27, ${\text{η}}_{\rho }^{2}$ = 0.06 or condition, *F*_(2.3,45.6)_ = 1.61, *p* = 0.21, ${\text{η}}_{\rho }^{2}$ = 0.07. In addition, there was no significant muscle × condition interaction, *F*_(3.3,65.3)_ = 0.74, *p* = 0.55, ${\text{η}}_{\rho }^{2}$ = 0.04. This confirmed that any facilitation in MEP amplitude could not be attributed to changes in coil positioning across experimental blocks.

### Main Analysis

**Figure 2:**
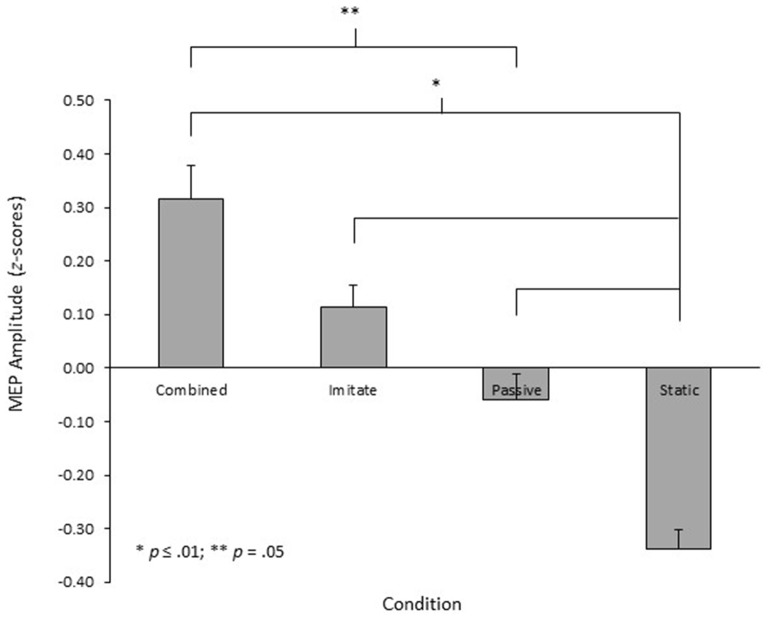
**Mean MEP amplitudes recorded from the right OP, ADM and FDI muscles in each of the four conditions of the experiment, displayed as *z*-scores**.

The 3 (muscle) × 4 (condition) repeated measures ANOVA performed on the *z*-score MEP amplitude data showed no significant main effect for muscle, *F*_(1.3,26.3)_ = 0.30, *p* = 0.65, ${\text{η}}_{\rho }^{2}$ = 0.02. There was, however, a significant main effect for condition, *F*_(3,60)_ = 14.5, *p* < 0.001, ${\text{η}}_{\rho }^{2}$ = 0.42 (see Figure 2). Pairwise comparisons with the Bonferroni adjustment showed that the MEP amplitudes in the three movement observation conditions were significantly larger than those obtained in the static hand condition (all *p* ≤ 0.01). There was no significant difference in MEP amplitude between the passive observation and observe with the intent to imitate conditions (*p* = 0.46), although the combined observation and imagery condition did produce significantly larger MEPs than the passive observation condition (*p* = 0.05). There was no significant difference in MEP amplitude between the combined observation and imagery and observe with the intent to imitate conditions (*p* = 0.65). Finally, there was no significant muscle × condition interaction, *F*_(3.1,62.3)_ = 1.36, *p* = 0.26, ${\text{η}}_{\rho }^{2}$ = 0.06.

## Discussion

The primary aim of this experiment was to establish whether different sets of viewing instructions provided prior to action observation would differentially facilitate corticospinal excitability. Specifically, the amplitudes of MEPs obtained during passive observation, observation with the intent to imitate, or combined observation and imagery of similar finger-thumb opposition movement sequences were compared against a control condition involving observation of a static hand. In line with the prediction for the first hypothesis, the results indicated that all three action observation conditions produced MEPs of significantly larger amplitude than were obtained during observation of the static hand. In partial support for the second hypothesis, the MEPs obtained during the combined observation and imagery condition were also significantly larger than those obtained during passive observation, but there was no difference between the amplitude of MEPs obtained in the observation with the intent to imitate and passive observation conditions. Differences in MEP amplitude between conditions are indicative of differences in corticospinal excitability (Rothwell, 1997; Naish et al., 2014). As such, these results indicate that whilst all three types of action observation facilitated corticospinal excitability in comparison to the control condition, the combined observation and imagery condition also resulted in greater facilitation of corticospinal excitability than occurred during passive observation.

The finding that action observation facilitated corticospinal excitability is likely indicative of activity in the hMNS, and is consistent with our first hypothesis and with a large body of previous research in this area (e.g., Fadiga et al., 1995; Strafella and Paus, 2000; Gangitano et al., 2001; Patuzzo et al., 2003; Borroni et al., 2005; Roosink and Zijdewind, 2010; Loporto et al., 2012; Williams et al., 2012). Although it is noteworthy that all three action observation conditions produced MEPs of larger amplitude than the control condition, the main finding of interest in this experiment was that combined observation and imagery of the movement sequence facilitated corticospinal excitability to a greater extent than passive observation. Importantly, the results of the ANOVA on the number of rejected MEPs data and the baseline EMG data immediately prior to the delivery of TMS indicate that this increased facilitation of corticospinal excitability during combined observation and imagery was not the result of increased EMG activity in this condition. Similarly, the results of the ANOVA on the pre-condition fixation cross data indicate that there were no significant differences in coil position between conditions. As such, the increased facilitation of corticospinal excitability found during combined observation and imagery can be attributed accurately to the experimental manipulation. This finding is consistent with an emerging body of TMS (e.g., Sakamoto et al., 2009; Ohno et al., 2011; Tsukazaki et al., 2012; Wright et al., 2014; Mouthon et al., 2015) and fMRI (e.g., Macuga and Frey, 2012; Nedelko et al., 2012; Villiger et al., 2013) research demonstrating greater activity in motor regions of the brain when observation is combined with simultaneous imagery, compared to passive observation. Traditional imagery interventions (i.e., imagery performed in the absence of observation) have been shown to activate the premotor cortex and other motor regions of the brain in a similar, although not identical, manner to observation (e.g., Grèzes and Decety, 2001; Jeannerod, 2001; Filimon et al., 2007; Munzert et al., 2008). Consequently, combining the two processes into a single intervention, in a “dual-simulation” approach, may cause the simulated action to resonate more strongly in the motor system of the observer (Vogt et al., 2013). The result of this may be reflected in the increased corticospinal excitability (e.g., Sakamoto et al., 2009; Ohno et al., 2011; Tsukazaki et al., 2012; Wright et al., 2014) and increased blood flow to cortical motor regions (e.g., Macuga and Frey, 2012; Nedelko et al., 2012; Villiger et al., 2013) found when observation and imagery are combined.

In contrast to the findings for combined observation and imagery, no differences in MEP amplitude were found between observation with the intent to imitate and passive observation. Despite the difference not being statistically significant, visual inspection of Figure 2 indicates a trend for larger MEPs in the observe to imitate condition, offering partial support for the findings of Roosink and Zijdewind (2010) whilst conflicting with the findings of Hardwick et al. (2012). This trend may be reflective of the strategies employed by participants to learn the movement sequence in the observe to imitate condition. Post-experiment debriefing sessions indicated that all participants used a mental rehearsal strategy to learn the movement sequence in the observe to imitate condition. Specifically, participants reported either numbering the fingers (*n* = 10) or naming the fingers (*n* = 9) and using internal self-talk to rehearse the sequence by either counting out or verbalizing the sequence whilst they watched the movement. The remaining two participants also reported using a mental rehearsal strategy in this condition, but were not able to explain how this was employed. In contrast, only five participants reported engaging in similar mental rehearsal strategies during the passive observation condition, with most participants reporting that they simply observed the hand as instructed and did not think about anything in particular. Where such rehearsal strategies were used during passive observation, they were reported to be more spontaneous and less effortful than were those used during the observe to imitate condition. Andres et al. (2007) have demonstrated that counting tasks facilitate corticospinal excitability in the hand muscles, reflecting the use of hand motor regions to monitor digits when counting in a sequence; presumably a developmental effect from childhood when hand movements play a crucial role in learning to count (Andres et al., 2007, 2008). Similarly, Oliveri et al. (2004) reported that processing of hand-action-related words facilitated corticospinal excitability in the hand muscles, indicating that processing of words associated with specific movements activates the motor areas involved in performing those movements. Taken together, these findings indicate that the additional numerical or verbal processing that participants engaged in during the observe to imitate condition, may have contributed to the non-significant trend for larger amplitude MEPs when participants observed the movement sequence with the intent to imitate, compared to when they observed passively.

Importantly, the additional verbal and numerical processing reported by participants in the observe to imitate condition was insufficient to elicit a significantly greater facilitation of corticospinal excitability in comparison to passive observation. In contrast, the additional processing of kinesthetic imagery during the combined observation and imagery condition did facilitate corticospinal excitability to a significantly greater extent than passive observation. Kinesthetic imagery has been shown to have a strong facilitatory effect on corticospinal excitability (Stinear et al., 2006). As such, the instruction for participants to simultaneously image the physiological sensations associated with the observed movement (e.g., the feelings of the finger muscles contracting and making contact with each other) during the combined observation and imagery condition may have produced stronger activity in the premotor and motor regions than occurred with the verbal or numerical processing used by participants in the observe to imitate condition. This may have contributed to the trend for larger amplitude MEPs in the combined observation and imagery condition compared to the observe to imitate condition, and resulted in the significantly greater facilitation of corticospinal excitability during the combined observation and imagery, compared to passive observation.

A somewhat unexpected finding in this experiment was that the facilitation of corticospinal excitability during the action observation conditions was found across all three muscles. A muscle-specific facilitation, whereby facilitation of corticospinal excitability is specific to the muscles that would be involved in performing the observed movement, has been reported in several TMS action observation experiments (e.g., Fadiga et al., 1995; Urgesi et al., 2006; Loporto et al., 2013). This effect, however, is not always reported for a variety of reasons including the stimulation timing and the number of muscles from which MEPs are recorded (for a review, see Naish et al., 2014). As all stimulations in this experiment were delivered at the point in the video where contact was made between the thumb and little finger, it was anticipated that facilitation of corticospinal excitability would only be detected in the OP and ADM muscles and not the FDI muscle. It is important to note, however, that the index finger was involved at certain points of the observed movement sequences albeit not at the time the stimulation was delivered. In addition, participants may have perceived the movements as whole hand actions rather than as several discrete movements, the muscles being in a state of isometric contraction during their abducted position when not moving and perceived as activity in the FDI. The fact that participants reported mentally rehearsing the whole sequence in the observe to imitate condition supports this contention. These two factors may have disrupted the muscle-specific facilitation effect and explain why facilitation of corticospinal excitability was also present in the FDI muscle.

Although outside the scope of the current study, there appear to be task-related differences in the effect that observe to imitate instructions have on corticospinal excitability. In TMS experiments where no additional facilitation of corticospinal excitability has been reported during observation with the intent to imitate in comparison to passive observation (e.g., Clark et al., 2004; Hardwick et al., 2012), participants observed familiar, previously learned, movement tasks. In contrast, where a significant facilitation of corticospinal excitability during observation with the intent to imitate has been reported in comparison to passive observation (e.g., Roosink and Zijdewind, 2010), participants observed a more novel finger tapping sequence. The trend for a facilitation of corticospinal excitability during observation with the intent to imitate in comparison to passive observation in the current experiment (see Figure 2), using a novel finger-thumb opposition sequence, provides partial support for this idea. It is possible, therefore, that there may be learning and task-related differences that influence the inhibitory-facilitatory effect during observation with the intent to imitate, and further research should explore this issue.

The results of this experiment indicate that combining action observation and imagery into a single strategy provides greater facilitation of corticospinal excitability than passive observation. Although this finding is important, there were a number of limitations to the experiment that should be acknowledged. Both the fact that experimental conditions were presented in a fixed order (i.e., static hand observation, passive action observation, observation with the intent to imitate, then combined observation and imagery) and the fact that a slightly different movement sequence was used for each experimental condition could be considered problematic. First, in relation to the issue of the fixed order, conditions were presented in this manner to reduce the likelihood of participants: (i) utilizing their observe to imitate strategy during passive observation trials and (ii) engaging in imagery during static hand, passive observation or observe to imitate trials. Despite this rationale, it is possible that the fixed order may have introduced order effects whereby familiarity with finger-thumb opposition movements or participant fatigue towards the end of the experiment may have influenced the amplitude of MEPs in the final experimental blocks. Second, the decision to use slightly different movement sequences for each experimental condition, was taken to ensure that participants were presented with novel movement sequences as familiarity with the observed sequence from an earlier condition may have influenced MEP amplitude in the final experimental blocks. Although care was taken to ensure that all movement sequences were of a similar nature (i.e., they all contained the same number of left or right directional movements and were all performed at the same speed) if certain movement sequences were perceived to be easier movements than others then MEP amplitude may have been influenced in these conditions. To alleviate these concerns, researchers could consider replicating this experiment with a between-participants design.

Given the fine motor movements used in this task, the findings from this experiment may have important implications for movement rehabilitation settings such as stroke recovery. Action observation interventions are increasingly recommended as movement therapy techniques that, if used as an adjunct to traditional physical therapies, could aid recovery of upper limb function in stroke survivors (Holmes, 2007; Holmes and Ewan, 2007; Ertelt and Binkofski, 2012; Sale and Franceschini, 2012; Small et al., 2012). Simulation interventions may contribute to improved motor function by promoting functional activity and plasticity in the damaged motor regions of the brain (Ertelt and Binkofski, 2012; Small et al., 2012) and increasing motivation to re-engage with a more active lifestyle (Holmes and Ewan, 2007). There is an emerging body of research supporting the efficacy of such interventions (e.g., Franceschini et al., 2012; Sugg et al., 2015). The current experiment has demonstrated that combined observation and imagery instructions facilitate corticospinal excitability to a greater extent than passive observation. These results suggest that in movement (re)learning settings, such as stroke rehabilitation, encouraging participants to imagine simultaneously performing the movement whilst they observe it may be a more effective simulation technique than observing passively. This proposal has received support from a recent behavioral experiment in healthy participants (Eaves et al., 2014), although the efficacy of such interventions for movement rehabilitation following stroke is yet to be established. In addition, in cases where imagery ability has been compromised following stroke (Ewan et al., 2010), there is evidence from sports research that combining observation and imagery may also help improve imagery vividness (Wright et al., 2015), although this suggestion is somewhat speculative at this stage. Future research should, therefore, seek to examine the efficacy of combined observation and imagery interventions for movement rehabilitation.

## Author Contributions

DJW, SAMcC, JW and PSH all contributed to the design of the experiment. DJW was responsible for participant recruitment, data collection, and data analysis. DJW, SAMcC, JW and PSH all contributed to the writing up of the manuscript. DJW was the lead author, with SAMcC, JW and PSH and contributing to different sections, reviewing drafts of the manuscript and making revisions to the manuscript.

## Conflict of Interest Statement

The authors declare that the research was conducted in the absence of any commercial or financial relationships that could be construed as a potential conflict of interest.
